# Supplementary material for: Metabolic reprogramming in Rheumatoid Arthritis Synovial Fibroblasts: A hybrid modeling approach
Source: PLoS Comput Biol. 2022 Dec 12;18(12):e1010408. doi: 10.1371/journal.pcbi.1010408 (PMC9779668; doi:10.1371/journal.pcbi.1010408)
Supplement: Attachment — Submitted filename: PLOS_Response_Reviewers.pdf [file pcbi.1010408.s014.pdf]

Reviewer 1:

In this manuscript, authors apply a combination of large-scale boolean models of signaling and gene regulation with flux balance analysis to deepen in the metabolic phenotype of Rheumatoid Arthritis Synovial Fibroblasts (RASFs). The manuscript is nicely written and structured, facilitating the reading and presenting the results in a coherent order. The proposed approach is appealing from the Systems Biology perspective, as authors attempt to understand the metabolic switch of RASFs combining available prior knowledge on the three main types of biological networks, namely signaling, gene-regulation and metabolism. Nevertheless, several of the assumptions made in this work need further support, and the proposed models present certain weaknesses that could be strengthened by addressing the points outlined below.

Major points:

We would like to thank the reviewer for their comments and the points raised. We provide point by point responses below.

Major Point 1: According to the first paragraph in the results section, the RA-map v2 contains 359 nodes, 14 inputs and 642 interactions that comprise signaling and gene regulatory interactions that are relevant in RA according to bibliographic evidence. These numbers fall short when compared to the whole number of gene expression changes that have been detected in previous analyses (check Lindberg et al., 2010). Authors should try to expand the underlying regulatory model content, and put it in the context of the total number of changes that have been detected in previous omics experiments.

Response to Major Point 1: The RASF model includes 359 nodes and 642 interactions covering signaling, gene regulation, and their impact on RASFs' metabolism. It is the Boolean analogue of the RA-map V2 (Zerrouk et al., 2022). The latter is the largest, fully annotated, mechanistic representation of existing knowledge related to the onset and progression of RA. It is based on the manual curation of 575 peer-reviewed articles and includes 720 species and 602 reactions. A key piece of information is that the interactions depicted in the map are not protein-protein interactions, inferred from interaction databases, but mechanistic, causal interactions based on experimental evidence. Moreover, the manual curation process allowed for disease and cell

specificity elevating the confidence of the included information. For more information about the RA-Map V2 construction, the reviewer can refer to Singh et al., 2020 and Zerrouk et al., 2022.

Considering the molecular interaction map and regulatory model's numbers in the context of the total number of changes identified in omics experiments can be challenging. A majority of said experiments in RA are performed on blood samples and are not RASF-specific. Even when samples are obtained from synovial tissue, as in Lindberg et al., 2010, it is not easy to conclude on their fibroblastic specificity (it could be macrophages). Also, a significant portion of identified differentially expressed components are not part of any known signaling or gene regulatory pathways in databases such as IPA, KEGG or WikiPathways. Even if they were identified as significantly expressed in RA, the lack of mechanistic information would make their wiring in the map and the associated model difficult.

Nevertheless, in omics experiments where criteria for disease and cell specificity were fulfilled, the biomolecules for which mechanistic information was available were added to the RA-map V2 and subsequently to the RASF model. For instance, addition of CXCL9, IRF7, and MYD88 in the RA-map V2 was based on the manual curation of Zhang et al., 2019's RASF-specific omics experiments' publication.

We have now added the following text to the manuscript to make our choice clearer to the reader (page 7, section 2.1.1. Inference of a Boolean model from the RA-map V2, lines 169-181):

"It is the largest, fully annotated, mechanistic representation of existing knowledge related to the onset and progression of RA. [...] The RA-map V2 is based on the manual curation of 575 peer-reviewed scientific publications and comprises 720 components and 602 reactions. It is an upgrade of the original global RA-map (Singh et al., 2020) with the addition of metabolic information and enrichment of signaling and regulatory pathways in RASF-specific knowledge. The latter were added from manual curation of omics experiments-associated publications. Additionally, the manual curation process allowed for disease and cell specificity elevating the confidence of the included information. Interactions depicted in the RA-map V2 are not protein-protein interactions, inferred from interaction databases, but mechanistic, causal interactions based on experimental evidence. For more information about the RA-Map V2 construction, please refer to Singh et al., 2020 and Zerrouk et al., 2022."

Major Point 2: Similarly, the context-specificity of the model towards RASF biology is based on information from the same underlying map. Since this is one of the main claims of this paper, and

given the dependence of other results on this assumption, authors need to provide additional evidence to support the cell-type specificity claim. Fortunately, there are two recent and extensive datasets where single-cell omics analyses were performed for synovial tissues in RA patients (Stephenson et al., 2018 and Zhang et al., 2019). Authors could use information from these datasets to support the initial conditions that they define and which theoretically transform the generic RA-map into a RASF specific map.

Response to Major Point 2: The RASF-specificity of our model was verified against several criteria and data. First of all, the construction of the RA-map V2 (Zerrouk et al., 2022) from the RA-map (Singh et al., 2020) was designed as RASF-specific. Only RASF-specific components and interactions were added, from manual curation of omics experiments-associated publications such as Zhang et al., 2019. In parallel, core compounds of the generic RA-map were further annotated based on RASF-specific scientific literature, increasing the overall RASF-specificity of the RA-map V2. However, the cellular specificity of RA-map V2 not being total, its translation into a model was restricted to pathways upstream and downstream of RASF-specific components demonstrated to be significant in RA's pathogenesis. The latter were identified from papers based on experimental evidence (see Table 1). Lastly, we used cell specific lists provided in the RA-Atlas, and more specifically the RASF-specific list, to calculate coverage and confirm the model's RASF specificity. Said list was obtained by aggregating single-cell omics data such as differentially expressed genes from GSE109449 (Mizoguchi et al., 2018), a single cell RNA-seq analysis of freshly isolated synovial fibroblasts in patients with RA along with curated data from low throughput RASF-specific data. The calculated percentage of RASF-specificity of our model is 82% (and 91% if we consider only exclusive components), which we consider a satisfactory percentage.

We have now added the following text to the manuscript (page 7, section 2.1.1. Inference of a Boolean model from the RA-map V2, lines 174-176) :
"It is an upgrade of the original global RA-map (Singh et al., 2020) with the addition of metabolic information and enrichment of signaling and regulatory pathways in RASF-specific knowledge. The latter were added from manual curation of omics experiments-associated publications."
We have now added the following text to the manuscript (page 8, section 2.1.2. Cellular-specificity assessment, lines 196-200):
"Lists are specific to RASFs, macrophages, synovial tissue, synovial fluid, blood and serum components, PBMC, and chondrocytes. First two are obtained by aggregating single-cell omics

data and literature data. Remaining sample-type specific lists are obtained through literature mining. Regarding RASFs, said omics data include differentially expressed genes from GSE109449 (Mizoguchi et al., 2018), a RASF-specific single cell RNA-seq analysis dataset.

Major Point 3: On the same line, authors employed a genome-scale metabolic model which is small when compared to any comprehensive and recent genome-scale metabolic model of human cells. An example is the Human-GEM described in Robinson et al., 2020, which contains 13069 reactions (in comparison to the 324 + 83 included in MitoCore) and 8366 metabolites (in comparison to 74 included in MitoCore). Why do authors employ such a small metabolic model? How do they know that the many metabolic events that are neglected by using such a small metabolic model are not relevant for the metabolic switch occurring in RASF? Authors need to provide further justification in the selection of the GEM selected.

Response to Major Point 3: We thank the reviewer for pointing this out. Human genome-scale metabolic models (GEMs) accounting for over 10,000 reactions have been published recently as the reviewer suggests and could actually be used within our hybrid modeling framework. Indeed, the primary aim of our work was to provide a proof of concept of a hybrid computational approach for coupling a knowledge-driven Boolean regulatory network to a metabolic network. Hence, our main concern was not in the computational aspects (FBA is not computationally costly) but rather in the reliability of both networks that would allow us to interpret results in a disease and cell specific context.

In the early stages of the project, we identified a few large GEMs (e.g. Recon 2.2 (Swainston et al., 2016), the Human Metabolic Reaction (Hongwu et al., 2007)) and spent time reflecting on the model we would use. We eventually opted for the MitoCore model for a variety of reasons presented below.

As described in Fritzemeier CJ, et al., 2017, non-realistic ATP production rates in several large GEMs' simulations (e.g. metabolic reconstructions of ModelSEED (Seaver et al., 2020) and MetaNetX (Moretti et al., 2016) databases) constitute their main limitation in our context. The MitoCore model, although smaller, has been demonstrated to correctly simulate core metabolism and predict physiologically realistic ATP production rates (Smith et al., 2017). Additionally, the gene-protein-reaction rules of large GEMs are often automatically reconstructed based on text mining and data analysis, not always demonstrating high accuracy. For instance, we were not able to confirm Recon 2.2's gene-protein-reaction associations in several cases (e.g. RRM1/N-

Formyl-L-Kynurenine association). Moreover, MitoCore is sufficient to account for our metabolic subsystems of interest: central carbon metabolism and energy production. It includes glycolysis, pentose phosphate pathway, citric acid cycle, electron transport chain, synthesis and oxidation of fatty acids, ketone body and amino acid degradation and covers all parts of central metabolism involved directly or indirectly with ATP production.
Therefore, we chose MitoCore, a smaller, but manually curated metabolic model verified against data from healthy and disease metabolism.
However, it is not negligible that other metabolic pathways and reactions could be identified with larger metabolic networks. As the performed metabolic analyses do not require particularly high computing power it can be adjusted to accommodate larger metabolic networks. One of our future steps would be to couple our regulation model to a larger GEM of human cells.

We have now added the following text to the manuscript (page 10, section 2.2 Metabolic model, lines 238-245):
"It [...] covers all parts of central metabolism directly or indirectly involved with energy production. This "core" model, although smaller in size compared to recently published human genome-scale metabolic models, allows to avoid many large genome-scale models' associated issues (e.g. unrealistic ATP production rates, automatic and not-curated reconstruction of improper gene-protein-reactions rules leading to incorrect compartmentalization of reactions or directionality constraints). Additionally, considering its manual curation, users can have great confidence in each reaction and have a better insight on the system's behavior, allowing for an easier evaluation of the results."
We have now added the following text to the manuscript (page 33, section 5. Perspectives, lines 688-691):
"Finally, the metabolic analyses performed within the framework do not require particularly high computing power, making them suitable for larger metabolic networks. Coupling the Boolean regulatory model with a larger human genome-scale metabolic model could be considered to expand identification of altered metabolic pathways in RASFs."

Major Point 4: Finally, authors need to demonstrate the advantages of their hybrid approach over other methods to achieve context-specific GEMs (see Hovratin et al., 2022). Using the single-cell information previously mentioned, authors can try to create a context specific GEM by setting an objective function that maximizes the overlap between enzymes that participate on selected

metabolic reactions and genes specifically expressed in RASF obtained from the single-cell data analysis.

Response to Major Point 4: Context-specific GEMs are based on the assumption that single-cell metabolism can be predicted from more easily measurable omics data such as mRNA. However, in order to parameterize such large models, massive amounts of omics data are needed. In this work, we propose a computational framework to derive a context-specific metabolic network from a knowledge-driven molecular interactions' Boolean model. Our framework's main strength lies in the reliability of our manually curated model, rather than a data-driven network, to address a lack of large-scale RASF-specific omics data. The previously cited issue of RA omics experiments' fibroblast specificity is also to be taken into account.

We have now added the following text to the manuscript (page 30, section 4. Discussion, lines 618-621):
"The main strength of this approach lies in the reliability of both manually curated models rather than data-driven networks. It allows to address a lack of large-scale RASF-specific omics data for the regulatory network's construction as well as improper automatic reconstructions for the metabolic network."

Minor points:

Minor Point 1: Given that two major points include the expansion and the creation of new context-specific GEMs, it is expected that the solution space will grow significantly, turning the model into a non-identifiable model. In this context, authors can apply DEXOM, a recent tool to explore alternative solutions in context-specific GEMs (see Rodriguez-Mier et al 2021).

Response to Minor Point 1: The goal of the work presented in this paper is not to build a context-specific GEM. It is rather to present a proof of concept for a hybrid modeling framework coupling a logical Boolean model to a metabolic network to derive additional metabolic constraints. The latter allows us to study the impact of gene regulation and signaling machineries on central metabolism in a cell and disease specific manner. In this context, the addition of the suggested tool does not align with the main scientific objective of the paper. Furthermore, from a purely

technical perspective, we propose a fully open-source, python-based pipeline. We aim to continue in the same spirit if we would need to integrate further steps in our pipeline.

Minor Point 2: A visualization of the trap states that occur during the regulatory model simulation would be beneficial for the understanding of the proposed approach.

Response to Minor Point 2: The complete trap-spaces of the RASF model are presented in Table 3 of the supplementary materials. A projection of the RASF model's trap-spaces on its ontological phenotypes (i.e. phenotypes included in the RASF model) has also been added in Table 3 (page 23) for a better understanding of the trap-spaces' biological interpretation (pages 22-24, section 3.2.1 Identification of regulatory trap-spaces, lines 465-516). Additionally, we added Figure 2, in the supplementary materials to facilitate the visualization of the proposed approach.

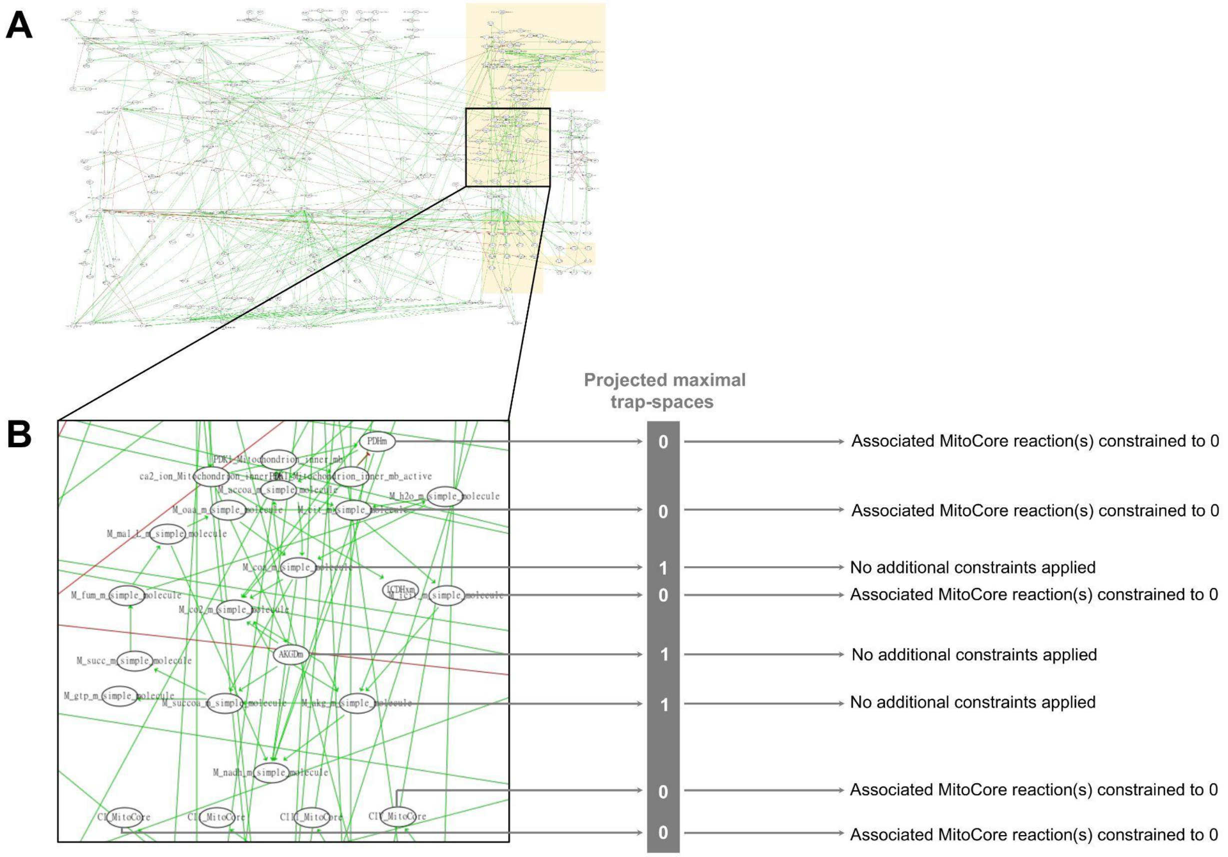

Figure S2. Visualization of the metabolic constraint extraction approach. **(A)** Visual representation of the RASF model with its metabolic components displayed in yellow. The regulatory components of the model are not considered in extracting additional metabolic constraints. **(B)** The metabolic reactions that are associated with metabolic components presenting maximal associated trap-spaces equal to 0 are constrained to 0 in the metabolic model. The reactions with maximal associated trap-spaces equal to 1 are not considered for extracting further l metabolic constraints.

Minor Point 3: For the sake of reproducibility, authors should deposit all the code and data needed to reproduce their results in a permanent archive like Zenodo (https://zenodo.org/) or figshare (https://figshare.com/), not only in GitLab.

Response to Minor Point 3: All data and code used to generate results are now available both in a GitLab repository (https://gitlab.com/genhotel/rasf-hybrid-model) and in a Zenodo permanent archive (https://doi.org/10.5281/zenodo.7181588). No deposit was made on Figshare, in the absence of data sets in our project.

We have now added the following text to the manuscript (page 34, section Availability, lines 715-716):
"All data and code used to generate results are available in a GitLab repository at https://gitlab.com/genhotel/rasf-hybrid-model and in a Zenodo permanent archive at https://doi.org/10.5281/zenodo.7181588 (both accessed on November 3rd 2022)."

Reviewer 2:

The authors present a hybrid modeling approach to analyse the rheumatoid arthritis synovial fibroblast (RASFs). They combine qualitative regulatory network with metabolic network and use the regulatory trap-spaces as constraints on the metabolic network. Their method is original and the integration of trap-spaces into FBA allows to propose a formal framework to the study the coupling of metabolic and regulatory networks. Their method allows to find expected experimental results such that the glycolytic switch.

The paper is suitable for the journal but there are some points which need to be explained:

Point 1: The integration of regulatory constraints in constraint-based metabolic models is not an obvious task. Recent works have begun to take into account both networks. The authors discuss about why they do not choice to use existing tools like flexflux (l.555) which is motivated by the difficulty to define initial conditions and intervals in flexflux. The authors propose to use trap-spaces to overcome these difficulties. However, the reader would like to be convinced about the biological interest of trap-spaces in the metabolism. Could the authors explain more about it ?

We would like to thank the reviewer for their comments and the points raised. We provide point by point responses below.

Response to Point 1: Trap-spaces are subspaces of state space that the system cannot escape. Each trap-space can contain one or several smaller trap-spaces. Minimal (or terminal) trap-spaces, i.e. trap-spaces which do not include smaller trap-spaces, offer a good approximation of attractors and can describe the asymptotic behavior of the system. Their analysis is fundamental as they can be linked to biological states, such as distinct cell fates or cellular phenotypes. Here, the trap-spaces of the RASF regulatory model reflect the impact of the RASF initial conditions on signaling and gene regulation that can further be linked to cellular metabolism.

We have now added the following text to the manuscript (pages 12-13, section 2.3.3. Projection of metabolic regulatory trap-spaces, lines 298-301):
"Said lists were used to project previously identified regulatory trap-spaces on the metabolic enzymes and metabolites. The latter reflected RASFs' signaling and gene regulation's impact on cellular metabolism, i.e. cellular phenotype."

Additionally, an extensive biological interpretation of all trap-spaces has been added in the new section 3.2.1 Identification of regulatory trap-spaces (see answer to Point 4).

Point 2: The trap-spaces are stable motifs which the system can not escape. However some asymptotic behaviors oscillate. How the authors manage with the oscillatory behaviors of the regulatory networks ?

Response to Point 2: As mentioned previously, minimal trap-spaces offer a good approximation of attractors and are totally able to capture dynamic oscillatory behavior. Said oscillatory trap-spaces would be represented by an asterisk and are not observed in our RASF model under RASF-specific conditions.
However, oscillatory trap-spaces could be observed for projected metabolic compounds or for regulatory components. Regarding metabolic compounds' associated trap-spaces, if they were to oscillate, they would not be taken into account within the hybrid modeling framework. Indeed, the latter only extracts constraints from metabolic compounds with a proven "inactive" asymptotic behavior (i.e. trap-spaces always equal to 0). As for non-metabolic regulatory components, oscillating trap-spaces can reflect biologically relevant feed-back loops or cycles and are accounted as a part of the model's dynamic complex behavior. They are not used within the hybrid modeling framework to extract additional metabolic constraints.

Point 3: The authors have to explain "minimal" trap-spaces line 251 ? what it means to calculate trap-spaces without performing simulation (line 257) ? Could they discuss about the computation time and the enumeration of trap-spaces ?

Response to Point 3: Minimal trap-spaces refer to trap-spaces which do not include smaller trap-spaces. The trap-spaces of a discrete dynamic system are identified without performing simulation, through a symbolic approach implementing a constraint-solving method. Their computation relies on the identification of positive and negative prime implicants for each component's function. In other words, their calculation is not based on dynamic analysis. In bioLQM (Naldi 2018), the tool used in our approach, trap-spaces can be obtained directly using binary decision diagrams or by using an ASP-based solver. For more information regarding trap-space identification, please refer to Zanudo, 2013 and Klarner, 2014.

We have now added the following text to the manuscript (page 12, section 2.3.2. Identification of regulatory trap-spaces, lines 277-286):

"Trap-spaces may overlap or include each other. Thus, minimal trap-spaces (later referred to as "trap-spaces" for readability), i.e. trap-spaces which do not include smaller trap-spaces, offer a good approximation of attractors and faithfully capture the asymptotic behavior of Boolean models. [...] Their computation relies on the identification of positive and negative prime implicants for each component's function without performing simulation but rather through a symbolic approach implementing a constraint-solving method (Zañudo & Albert, 2013; Klarner et al., 2014). In bioLQM (Naldi 2018), trap-spaces can be obtained directly using binary decision diagrams or an ASP-based solver."

Point 4: They show 8 Trap-spaces in the supplementary information but the reader has difficulty understanding their biological meanings. Do they represent a specific phenotype profile ? What differentiates them ?

Response to Point 4: We would like to thank the reviewer for raising this important point. Indeed, a biological interpretation of the eight different trap-spaces was lacking and has now been added to the manuscript in the new section 3.2.1 Identification of regulatory trap-spaces (pages 22-24, lines 465-515):

"Eight different trap-spaces were identified, each trap-space reflecting a different subspace of RASFs' cellular phenotypes. Most components' values are stable within all trap-spaces (always fixed either at 0 or 1) but others vary within trap-spaces. If we restrict the projection of the trap-spaces to the nine RASF model's ontological phenotypes, we obtain the results presented in Table 3. Ontological phenotypes refer to nodes representing a distinct cellular outcome in the RASF model.

Table 3. Projection of the RASF model's trap-spaces on its nine ontological phenotypes.

Ontological phenotype	Trap-space							
	0	1	2	3	4	5	6	7
Angiogenesis	1	1	1	1	1	1	1	1
Apoptosis	0	0	0	0	0	0	0	0

Bone Erosion	1	1	1	1	1	1	1	1
Cell Chemotaxis, Recruitment, Infiltration	1	1	1	1	1	1	1	1
Cell Growth, Survival, Proliferation	1	1	1	1	1	1	1	1
Hypoxia	1	0	1	0	0	0	1	1
Inflammation	1	1	1	1	1	1	1	1
Matrix Degradation	1	1	1	1	1	1	1	1
Osteoclastogenesis	1	1	1	1	1	1	1	1

As observed in Table 3, the phenotypes of angiogenesis, bone erosion, cell chemotaxis, cell growth, inflammation, matrix degradation and osteoclastogenesis exhibit an asymptotic stable active state when the model is simulated under RASF-specific conditions. These results are consistent with known RASFs' biological aggressive behavior as described in scientific literature (Bartok, Firestein 2011).

The asymptotic state of ontological phenotypes is the result of the combined regulation exerted by their upstream regulators, as described in the logical formulas. Thus, the behavior of biomarker groups associated with the ontological phenotypes can be identified in the different trap-spaces. Subsequently, this behavior can be used for comparison against experimental evidence.

For instance, all interleukines (e.g. IL121, IL18, IL1B, IL33, IL6) being active under RASF-specific conditions accounts for the asymptotic active state of the ontological inflammatory phenotype and confirms their experimentally observed function of inflammation drivers (Magyari et al., 2014).

Likewise for the majority of the matrix metalloproteinases (e.g. MMP3, MMP9, MMP13) leading to matrix degradation (Burrage et al., 2006) or cytokines (e.g. TNF, IL17) activating bone erosion and osteoclastogenesis (Schett, Gravallese 2014).

A pattern of growth factors' (e.g. PDGFA, FGF1, VEGFA) activation is observed within the eight different trap-spaces and is associated with activation of the cell growth and proliferation phenotype. These findings are consistent with experimental evidence indicating them as key factors (Rosengren et al., 2016, Harada et al., 1998).

This proliferative behavior is confirmed in parallel by an asymptotic inactive state of the apoptotic phenotype, reproducing fibroblasts' resistance to programmed cell death in RA (Baier et al., 2003). Accordingly with biological knowledge, it is due to the active state of anti-apoptotic components (e.g. CAV1) and the inactivity of pro-apoptotic ones (e.g. Bak, Bax) in all eight trap-

spaces under RASF-specific conditions.
Finally, the hypoxic phenotype varies within trap-spaces, reflecting a biologically relevant feed-back loop. Trap-spaces where hypoxia is active are associated with active HIF1 and inactive PHD2. On the contrary, trap-spaces where hypoxia is inactivated are associated with inactive HIF1 and active PHD2. It reflects the well-known regulation of the cellular response to hypoxia by PHD2 through HIF1 (Berra et al., 2003).
The variations of fixed values within the eight trap-spaces can also be interpreted at the level of the RASF model's regulatory pathways. For instance, in trap-spaces 0, 1, 5, and 7, MAPK1 is active in parallel with BCL2. The latter are inactive in trap-spaces 2, 3, 4, and 6. It reflects the regulation of BCL2 through the MAPKs pathway (Trisciuoglio et al., 2005)."

Point 6A: The figure 5 shows the main active pathways of the ccm however, the authors test 14 initial conditions. How were these conditions chosen?

Response to Point 6A: We performed an extensive literature search to identify RASF specific initial values for all 14 inputs of the regulatory model and to two intermediate nodes (ATP and HIF1) to reproduce RASF-specific conditions (see Table 2). Here, with the term input we refer to extracellular ligands that do not have any upstream regulators. Each initial value was based on the analysis of omics experiments in relevant scientific publications. The control of these 16 components over the model's dynamics is proved by the results of value propagation where the RASF-specific conditions allowed us to fix the value of 313 compounds out of 359.

We have now added the following text to the manuscript (page 9, section 2.1.4. Validation of the regulatory model's behavior, lines 224-227):
"Initial values are assigned to all regulatory model's inputs (i.e. extracellular ligands which do not have any upstream regulators) and a few intermediate nodes to reproduce RASF-specific conditions. Inputs are suspected to exert a significant control on the model's dynamics due to the linearity of signal transduction in the RA-map V2 and associated model."
Table 2 was also modified to highlight the regulatory model's inputs.

Point 6B: In all trap-spaces of the RASF model, the PDHm = 0 which necessarily prevent to use the OXPHOS and thus lead a glycolytic profile to produce ATP. Could the authors discuss their initial choice and the link with the enzyme trap-spaces ?

Response to Point 6B: Regarding PDHm, all its trap-spaces being equal to 0 under RASF-specific initial conditions is not an imposed constraint on the RASF model to prevent it from using OXPHOS. It is rather the outcome of the complex RASF-specific regulatory effect on PDHm. Indeed, when exporting its entire upstream regulatory network from the RASF model, we obtain a Boolean model of 170 compounds and 299 interactions. Thus, in said RASF-specific conditions and according to the regulatory model's Boolean rules, PDHm's behavior is the dynamic combinatorics of all the latter.

Additionally, although PDHm's projected trap-spaces in RASF-specific conditions, as well as in all regulatory variants except C3, being always equal to 0 could suggest that inhibiting its associated reaction may have a key role in metabolic reprogramming, it is not sufficient. Indeed, when running a FBA on MitoCore's network after only constraining R_PDHm (i.e. the reaction catalyzed by PDHm) to 0, the proportion of ATP produced through glycolysis is 0.0414, similar to the healthy profile proportion and far from the RASF-specific altered profile where it is 0.85.

Point 7: Figure 4B is not readable, what should we understand ?

Response to Point 7: Figure 4B illustrated the projection of the value propagation results onto the regulatory network. However, it was indeed not very readable and probably misleading as value propagation is only an intermediate step of our hybrid modeling framework. We have decided to remove the figure from the manuscript.
